# Outcome and Safety after 103 Radioembolizations with Yttrium-90 Resin Microspheres in 73 Patients with Unresectable Intrahepatic Cholangiocarcinoma—An Evaluation of Predictors

**DOI:** 10.3390/cancers13215399

**Published:** 2021-10-27

**Authors:** Karolin J. Paprottka, Franziska Galiè, Michael Ingrisch, Tobias Geith, Harun Ilhan, Andrei Todica, Marlies Michl, Jonathan Nadjiri, Philipp M. Paprottka

**Affiliations:** 1 Department of Diagnostic and Interventional Neuroradiology, Technical University Munich, 81675 Munich, Germany; karolin.paprottka@tum.de; 2 Department of Clinical Radiology, LMU—University of Munich, 81377 Munich, Germany; Franziska.Galie@med.uni-muenchen.de (F.G.); michael.ingrisch@med.uni-muenchen.de (M.I.); 3 Department of Interventional Radiology, Technical University Munich, 81377 Munich, Germany; tobias.geith@tum.de (T.G.); jonathan.nadjiri@tum.de (J.N.); 4 Department of Nuclear Medicine, LMU—University of Munich, 81377 Munich, Germany; Harun.Ilhan@med.uni-muenchen.de (H.I.); andrei.todica@med.uni-muenchen.de (A.T.); 5 Department of Oncology, LMU—University of Munich, 81377 Munich, Germany; Marlies.Michl@med.uni-muenchen.de

**Keywords:** intrahepatic cholangiocarcinoma, radioembolization, survival, outcome, safety, repetitive TARE

## Abstract

**Simple Summary:**

TARE with yttrium-90 (90Y) resin microspheres is emerging in many countries as a treatment option for ICC. Identification of patients that will benefit from TARE is a clinically relevant problem with individual but also economical relevance. The aim of this study was to detect outcome predictors for patients with ICC after TARE with 90Y resin microspheres. We found TARE with 90Y resin microspheres to be a safe treatment option for unresectable ICC. Predictive factors for TARE in ICC are CA-19-9 response, tumor burden, and cholinesterase. Multiple TARE sessions might further improve overall survival.

**Abstract:**

Trans-arterial radioembolization (TARE) is increasingly evaluated for unresectable intrahepatic cholangiocarcinoma (ICC). Not all ICC patients benefit equally well from TARE. Therefore, we sought to evaluate variables predicting progression-free survival (PFS) and overall survival (OS). Patients with non-resectable ICC underwent TARE and were treated with 90Y resin microspheres. Baseline characteristics, biochemical/clinical toxicities, and response were examined for impact on PFS and OS. A total of 103 treatments were administered to 73 patients without major complications or toxicity. Mean OS was 18.9 months (95% confidence intervals (CI); 13.9–23.9 months). Mean and median PFS were 10.1 months (95% CI; 7.9–12.2) and 6.4 months (95% CI; 5.20–7.61), respectively. Median OS and PFS were significantly prolonged in patients with baseline cholinesterase (CHE) ≥ 4.62 kU/L (OS: 14.0 vs. 5.5 months; PFS: 6.9 vs. 3.2 months; *p* < 0.001). Patients with a tumor burden ≤ 25% had a significantly longer OS (15.2 vs. 6.6 months; *p* = 0.036). Median PFS was significantly longer for patients with multiple TARE cycles (24.4 vs. 5.8 months; *p* = 0.04). TARE is a considerable and safe option for unresectable ICC. CA-19-9, CHE, and tumor burden have predictive value for survival in patients treated with TARE. Multiple TARE treatments might further improve survival; this has to be confirmed by further studies.

## 1. Introduction

Intrahepatic cholangiocarcinoma (ICC) is a rare disease with approximately 3000 cases diagnosed every year in the USA [1,2]. ICC is the second most common primary liver cancer and accounts for approximately 10–20% of all primary liver tumors [3]. ICC is often clinically silent in its early stages, and patients are diagnosed when surgery is no longer possible. As a consequence, prognosis is poor (5-year survival < 5%). Surgical treatments (liver transplantation or complete surgical resection) are currently the only potentially curative therapy. Nevertheless, only 20% of patients with ICC are eligible for resection due to anatomic location, disease spread, inadequate hepatic reserve, or limiting comorbidities [4,5,6]. Median survival for patients with untreated unresectable ICC has been reported to be between 3–6 months [6,7]. It is of note that survival for liver-only ICC is relevantly different from other bile duct cancers [8].

Based on a phase III trial, ABC-02 cisplatin-gemcitabine is the standard of care for ICC [9]. Still, median survival is <1 year [9]. Interventional therapy aims to control local tumor growth, relieve symptoms, and improve and preserve quality of life. Locoregional therapies, including trans-arterial chemoembolization (TACE) or radiofrequency ablation (RFA) have been proposed [10]. Despite their role in the therapy regimen of hepatocellular cancer (HCC), they are currently not the standard of care in locally advanced ICC [11]. Due to the relative radio-sensitivity of the liver, external beam radiation therapy (EBRT) has a limited role in the treatment of liver malignancies [12]. Trans-arterial radioembolization was effective in unresectable HCC and liver-dominant metastasis of neuroendocrine tumors (NET) [13], colorectal cancer (CRC), and metastatic breast cancer (MBC) [13,14]. Therefore, as ICC and HCC are generally radiosensitive, TARE with yttrium-90 (90Y) resin microspheres is emerging in many countries as a treatment option for ICC [13,15,16,17]. However, the response to TARE in ICC can be heterogeneous for various reasons. Further, identification of patients that will benefit from TARE is a clinically relevant problem with individual but also economical relevance [18].

The aim of our study was to detect outcome predictors for patients with ICC after TARE with 90Y resin microspheres (SIR-Spheres; Sirtex Medical Ltd., Sydney, Australia).

## 2. Materials and Methods

### 2.1. Patient Selection

We retrospectively analyzed 73 consecutive patients (103 TARE procedures) with unresectable ICC who were treated with 90Y resin microspheres.

Inclusion criteria were defined as follows: non-resectable ICC determined in an interdisciplinary tumor board; absence of significant extrahepatic disease (EHD) (patients with stable intra-abdominal lymph nodes were included, progression of lymph nodes or other kind of metastases was considered significant); failure to respond to other types of medical, surgical, or local ablative treatment modalities; no portal vein occlusion; adequate biochemical and hematological function (total bilirubin < 2 mg/dL, albumin and pseudocholinesterase within normal range, and sufficient coagulation); no relevant comorbidities; and written, informed consent. The study was approved by the local ethics committee.

### 2.2. Dosimetry for Radioembolization

The dose was calculated with the modified body surface area (mBSA) method [19]. mBSA was recommended by a consensus report as the most appropriate method avoiding the rare occurrence of radioembolization-induced liver disease (REILD).

### 2.3. Radioembolization Procedure

Within a month before TARE, patients underwent hepatic angiography with application of 100 MBq of 99mTc-macroaggregated albumin (MAA) to map the hepatic arterial tree and to detect and occlude relevant aberrant vessels where necessary using micro-coil embolization, and to calculate the shunt fraction of labeled microspheres to the lung.

During a subsequent hepatic arterial catheterization, 90Y resin microspheres suspended in water for injection were injected under intermittent fluoroscopic visualization, alternating with a contrast medium to preserve antegrade hepatic arterial flow. The prescribed activity was administered either in the whole liver, lobe, or as a sequential lobar treatment, according to tumor burden [20]. Within 24 h of therapy, single photon emission computed tomography scans (Bremsstrahlungs SPECT/CT) were performed to confirm target deposition of microspheres.

### 2.4. Follow-Up: Response, Toxicity, Survival

After TARE, tumor response was assessed by contrast-enhanced computed tomography (CT), magnetic resonance imaging (MRI), or PET-CT using the Response Evaluation Criteria in Solid Tumors (RECIST) [21]. Response was evaluated by two experienced radiologists. Up to five lesions were measured. Minor differences in quantification of the diameters of the lesions did not lead to differences in the tumor response scoring.

Pre- and post-treatment laboratory tests included liver function tests (bilirubin, albumin, serum cholinesterase [CHE], thrombocytes, C-reactive protein [CRP], and tumor marker [CA 19-9]). Patients remained in the hospital for 4–5 days after TARE with daily clinical and laboratory evaluation. In addition, laboratory tests and imaging were performed after 3 months. Acute and late adverse events were recorded at each visit during follow-up. Classification of adverse events was performed according to common terminology criteria for adverse events (CTCAE) version 5.0 [22]. Telephone follow-up with the referring physicians or the patients was used to capture survival data.

### 2.5. Statistical Analyses

Continuous variables are presented as means, medians, and interquartile range, and categorical variables as frequencies and percentages. Median progression-free survival (PFS) was calculated in months between the date of the first TARE until radiological progression or all-cause death, whichever occurred first. Median overall survival (OS) was calculated in months from the date of the first TARE until all-cause death. PFS and OS were evaluated using the Kaplan-Meier method and the univariate Cox proportional hazard method. Between-group differences in PFS and OS were assessed with log-rank tests for the variables age, sex, tumor burden, tumor response according to RECIST, number of TARE cycles, CA-19-9 response status, and baseline levels of bilirubin and CHE; CA-19-9 response was deemed to be achieved with a ≥30% decrease at 3 months compared with baseline value of CA-19-9.

The level of significance was set at *p* ≤ 0.05. The software package IBM SPSS Statistics for Windows, Version 20.0, released 2011 (IBM Corp., Armonk, NY, USA) was used.

## 3. Results

Seventy-three patients (33 women, 40 men, mean age of 64.5 (range 29.7–91.8 years)) with histologically confirmed unresectable ICC) underwent TARE as described above (Table 1).

The median total activity of 90Y resin microspheres delivered was 1.5 GBq (range, 0.4–2.5 GBq). A total of 103 treatment sessions were performed, including 25 single-session whole-liver administrations, 25 one-lobe single-session treatments, 17 (=34 TARE sessions) whole-liver treatments in two sessions and 6 cases with more than two treatment sessions (Table 2). Patients with multiple treatments underwent on average 2.2 sessions. Median time between the repetitive TARE cycles was 9.6 months (294 days).

### 3.1. Outcome—Treatment Response (RECIST)

Partial response (PR), stable disease (SD), and progressive disease (PD) were observed after 3 months in 18 (25%), 36 (49%), and 19 (26%) patients, respectively.

### 3.2. Outcome—Survival

**Figure 1:**
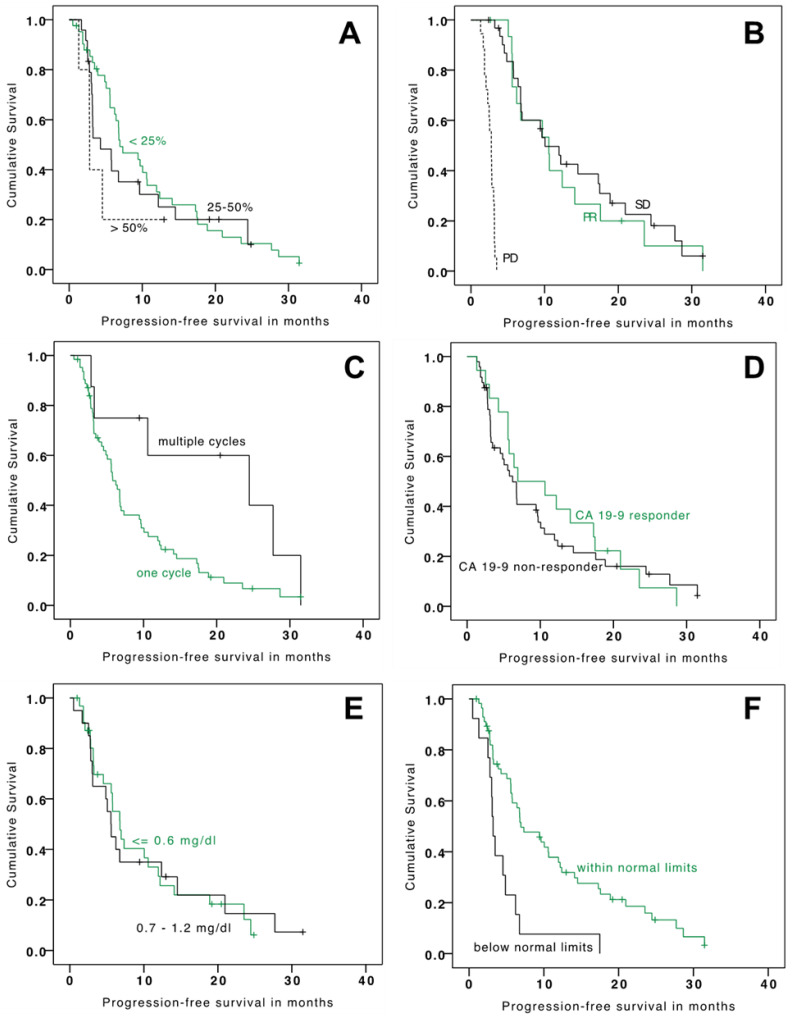
Kaplan-Meier analysis for progression-free survival stratified by tumor burden at baseline (**A**), tumor response (**B**), number of treatment cycles (**C**), CA19-9 response (**D**), baseline levels of bilirubin (**E**), and baseline levels of cholinesterase (**F**).

**Figure 2:**
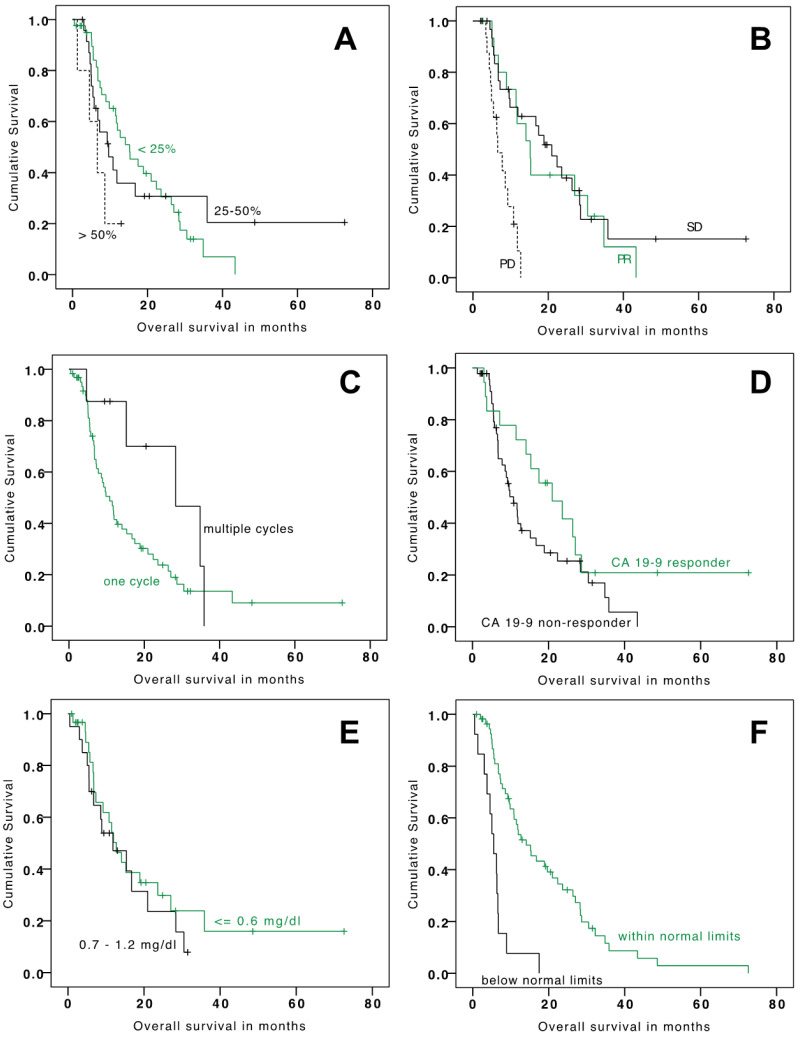
Kaplan-Meier analysis for overall survival stratified by tumor burden at baseline (**A**), RECIST response (**B**), number of treatment cycles (**C**), CA19-9 response (**D**), baseline levels of bilirubin (**E**), and baseline levels of cholinesterase (**F**).

The mean and median OS since TARE were 18.9 months (95% CI: 13.9–23.9) and 11.8 months (95% CI: 7.3–16.3), respectively (Table 3). Mean and median PFS were 10.1 months (95% CI: 7.9–12.2) and 6.4 months (95% CI: 5.2–7.6), respectively (Table 3). Factors that significantly prolonged median OS included tumor burden ≤ 25% at baseline versus tumor burden > 50%, a PR versus PD at 3 months, and baseline CHE ≥ 4.62 kU/L versus CHE < 4.62 kU/L (Table 3 and Figure 1). Factors that significantly prolonged median PFS included a PR versus PD at 3 months, baseline CHE ≥ 4.62 kU/L (lower limit of normal values) versus CHE < 4.62 kU/L and multiple treatment cycles versus one treatment cycle (Table 3 and Figure 2). It is of note that patients with repetitive TARE-sessions had a relevant prolonged overall survival (28.4 vs. 10.8 months). Progression-free survival was also relevantly better for patients with repetitive TARE (24.4 vs. 5.8 months).

Overall, 20 patients (27%) had a CA-19-9 response. A trend towards improved OS was observed for patients with a CA-19-9 response compared with those without a response (Table 3). At discharge CHE, albumin and thrombocyte levels decreased from baseline and subsequently increased (thrombocyte levels showed further decline) after 3 months but remained below baseline levels (Table 4). Bilirubin and CRP levels increased from baseline to time of discharge (Table 4). From discharge to month 3, the bilirubin value remained stable, whereas CRP decreased to levels closer to baseline (Table 4).

### 3.3. Safety

The procedure was associated with an acceptable toxicity profile (<Grade 4; no life-threatening events) including nausea and pain being the most frequent adverse events. No REILD was noted (Table 5). No adverse events attributed to the interventions themselves were observed.

## 4. Discussion

The main findings of this study are as follows: (i) trans-arterial radio-embolization (TARE) appears to improve overall survival in patients with irresectable intrahepatic cholangiocarcinoma (ICC) compared to the reported survival with standard care in the literature; (ii) CA-19-9, cholinesterase, and tumor burden have predictive value for survival in those patients treated with TARE; (iii) Multiple TARE treatments might further improve survival.

### 4.1. Outcomes, Previous Results, and Predictors

Effective treatment of irresectable ICC is clinically challenging, resulting in increased interest in TARE therapy in these patients [15,17,18,20]. Results from those studies highly implicate improved survival for patients treated with TARE. The results of our study exhibit an improved mean survival compared to standard care, which is also in line with previously reported outcomes [6,7,20]. Although current data suggest that TARE is a considerable treatment option, identification of patients that benefit from this specific treatment can be complicated. Previous papers have reported tumor burden as a predictive parameter in patients with TARE and ICC [16]. Similarly, in our study tumor burden was highly predictive and further cholinesterase exhibited good predictive value. Cholinesterase can be considered to be an inverse surrogate for tumor burden but also for the amount of functioning liver parenchyma. Previous chemotherapy, resection, ablation, or liver disease might also have reduced the amount of functioning parenchyma. As a result, doses for TARE might require relevant reduction below effective treatment levels to avoid radioembolization-induced liver disease, possibly also explaining the dependency of cholinesterase and survival with TARE next to other confounders. We observed improved survival (overall survival and progression-free) in patients with multiple TARE and sequential treatments for the same ICC lesions indicating a possible increased effectiveness of aggressive and sequential TARE treatment strategy. This finding has not been reported before and the effects are quite relevant in our study. However, we should not exclude the possibility that this effect is a result of the absence of treatment alternatives in the study population leading to increased numbers of TARE sessions in patients with longer survival. The causality remains somewhat unclear; patients with better survival might be treated more often because of their longer survival.

We further confirm the response of CA19-9 to prior chemotherapy to be a relevant predictor for survival in patients with ICC and TARE, which is comparable to results published before but in standard-care patients without TARE [23]. However, it can be speculated that CA19-9 response itself indicates, e.g., a certain genetic subtype of the tumor that is more susceptible to cytotoxic treatments in general and thus indicates whether the tumor might respond to TARE. In addition, tumor response according to RECIST criteria after 3 months was a factor associated with better survival. Other factors included in our analysis, such as patient age, sex, or bilirubin levels did not have an impact on patient outcome.

In this study, decreased CHE levels below reference values is a negative predictor for both OS and PFS. This is based on the assumption that CHE is a negatively correlated surrogate for the tumor burden. Large tumor masses with extensive liver infiltrations can impair liver function and as a consequence lead to a decrease in the unspecific liver synthesis parameter CHE.

### 4.2. Safety of TARE in ICC

Mouli et al. [24] reported about 46 patients (35% and 15% with previous chemotherapy or liver-directed therapies) who underwent TARE with glass spheres. Disease control (WHO imaging criteria) was reported in 98% of all treated patients, and >50% necrosis on follow-up imaging (EASL guidelines) in 73% of patients. Major post-treatment complications were fatigue (54%) and abdominal pain (28%), which is in line with our and other studies about TARE for hepatic tumors [13,25]. In this present study we observed no procedural complications; no life-threatening adverse events occurred (<Grade 4; no life-threatening events).

### 4.3. Treatment Alternatives

Other minimally invasive treatment options including RFA and TACE have been investigated, and RFA is known to be an effective treatment for focal liver lesions [26]. A recent study investigating RFA in ICC reports promising results and also prolonged survival compared to standard care [27]. In comparison to our results with TARE, complication rate and gravidity of the complications was higher for RFA. Several studies differing in quality have examined the use of TACE for patients with unresectable ICC [28,29,30]. In the prospective study of Kiefer et al. [28] with 62 ICC patients, PR, SD, and PD according to RECIST were reported in 11%, 64%, and 24% of patients, respectively. Five patients suffered from major complications. Post-embolization syndrome (CTCAE grade 1 or higher of post-procedural pain, fever, nausea, or vomiting) occurred in 65% of all patients. Median OS and median time to progression (TTP) after first chemoembolization were 15 months and 8 months, respectively. The potential procedural risk from catheterization for patients with TACE and TARE are similar. In comparison, per-session costs for TACE are considerably lower compared to TARE. However, TACE is not eligible for intermediate to increased tumor burden or diffuse tumor distribution.

TARE could be considered as an alternative to TACE in patients with increased tumor burden or in patients not suited to TACE [25].

A recent systematic review compared outcomes of different treatment methods in ICC. Even though the quality of available data is limited, ablation seems to be the best option in unresectable lesions. However, not all lesions are eligible for ablation. In tumors that are large or unfavorably located for ablation, trans-arterial therapies seem to play an important role [31].

Edeline et al. demonstrated improved survival for patients in ICC for a combination of TARE and chemotherapy in their prospective study and concluded that this combination is a valuable treatment option. This underlines that improved survival for patients with ICC requires an interdisciplinary and elaborated treatment strategy involving interventional radiologists, oncologists, and surgeons [17].

### 4.4. Limitations

This was a retrospective single-center study. As many patients received therapies before TARE (71% treated with chemotherapy, 31% treated with surgery, and 7% treated with RFA or TACE), attribution of effect sizes might by impaired. Subgroup analyses of OS and PFS for patients with different previous treatments might yield different or further results but has been waived because of the small sample sizes in the subgroups.

## 5. Conclusions

TARE with 90Y resin microspheres is a safe treatment option for unresectable ICC. Predictive factors for TARE in ICC are CA-19-9 response, tumor burden, and cholinesterase. Multiple TARE sessions might further improve overall survival; this has to be confirmed by further studies. Prospective randomized trials comparing TACE, TARE, RFA, and systemic chemotherapy will be necessary to further improve therapy for unresectable ICC.

## Figures and Tables

**Table 1:** Baseline characteristics.

Variable	No. of Patients	%
Total cohort	73	100
Age		
<65 years	31	42
≥65 years	42	58
Male	40	55
Previous therapies		
Surgery	23	32
Radiofrequency ablation	1	1
Prior trans-arterial chemoembolization	4	5
Prior chemotherapy	52	71
Tumor characteristics		
Extra-hepatic disease	37	51
Tumor burden		
≤25%	44	60
26–50%	24	33
>50%	5	7
Laboratory data		
Bilirubin (absolute)		
≤0.6 mg/dL	44	60
0.7–1.2 mg/dL	29	40
Cholinesterase (absolute)		
Within normal limits (≥4.62 kU/L)	59	81
Below normal limits (<4.62 kU/L)	14	19

**Table 2:** Location of radioembolization and dosimetry (MBq).

Treatment Concept (Patients)	*n* (%)	Calculated Dose		Applied Dose	
		Median Dose	Interquartile Range	Median Dose	Interquartile Range
Overall	73	1500	933–1940	1373	881–1848
Location					
One lobe, single session	25 (34%)	1067	412–2500	1050	404–2157
Whole liver, single session	25 (34%)	1960	750–2500	1887	740–2475
Whole liver, two sessions	17(23%)	1000	400–1500	1009	390–1512
Multiple treatments	6 (8%)	966.5	560–2000	1000.5	564–1874

**Table 3:** Median OS and PFS in months.

Category	*n* (%)	Overall Survival (Months)			Progression Free Survival (Months)		
		Median	95% CI	*p*-value	Median	95% CI	*p*-value
All patients		11.82	7.32–16.32		6.41	5.20–7.61	
Tumor burden		11.83	7.32–16.32		6.40	5.2–7.61	
≤25%	44 (60%)	15.21	8.86–21.56	(reference)	6.93	3.67–10.19	(reference)
26–50%	24 (33%)	9.62	4.54–14.70	0.963	4.27	0.34–8.19	0.515
>50%	5 (7%)	6.60	2.16–11.04	0.036	2.79	2.72–2.86	0.143
CA-19-9 response		11.95	7.53–16.38		6.73	5.58–7.88	
Yes	20 (27%)	20.96	10.44–31.48	(reference)	6.93	0.0–15.74	(reference)
No	53 (73%)	10.81	7.74–13.88	0.098	6.21	4.38–8.04	0.654
RECIST response		11.82	7.32–16.32		6.40	5.20–7.60	
Partial response	18 (25%)	15.21	10.64–19.77	(reference)	10.59	5.68–15.47	(reference)
Stable disease	36 (49%)	20.96	13.55–28.37	0.637	10.05	6.54–13.56	0.634
Progressive disease	19 (26%)	6.60	3.99–9.21	<0.001	2.76	2.28–3.23	<0.001
Bilirubin		11.46	7.94–14.98		6.20	4.67–7.74	
<0.6 mg/dL	44 (60%)	11.95	8.75–15.16	(reference)	6.76	4.83–8.69	(reference)
0.7–1.2 mg/dL	29 (40%)	8.903	4.19–13.61	0.419	5.55	4.40–6.70	0.967
Cholinesterase		10.87	7.88–13.86		6.40	5.08–7.73	
≥4.62 kU/L	59 (81%)	14.0	8.59–19.59	(reference)	6.93	3.56–10.30	(reference)
<4.62 kU/L	14 (19%)	5.52	3.51–7.52	<0.001	3.22	2.68–3.76	0.001
Treatment cycles		11.83	7.33–16.30		6.41	5.20–7.61	
One	67 (92%)	10.81	7.78–13.84	(reference)	5.78	4.69–6.88	(reference)
Multiple	6 (8%)	28.35	9.74–46.97	0.156	24.44	0.0–52.067	0.04

CI = Confidence interval; RECIST = response valuation criteria in solid tumors.

**Table 4:** Laboratory values at baseline, discharge, and 3 months after radioembolization.

Laboratory Value	Baseline		Follow-Up (Discharge)				Follow-Up (3 Months Post Radioembolization)			
	Median	IQR	Median	IQR	Change from Baseline		Median	IQR	Change from Baseline	
					Abs.	Rel. in %			Abs.	Rel. in %
Bilirubin (mg/dL)	0.60	0.5–0.8	0.7	0.55–1.0	0.1	11.81	0.7	0.6–1.2	0.1	25
Serum cholinesterase (kU/L)	6.62	5.27–7.58	5.79	4.55–6.85	−0.85	−12.8	5.90	4.54–6.89	−0.87	−11.03
Albumin (g/dL)	4.25	4.0–4.5	3.8	3.5–4.0	−0.40	−9.64	4.1	3.3–4.4	−0.2	−4.13
Thrombocytes (Thousand/µL)	197	150.5–253.0	160.0	114.0–197.5	−39.0	−22.06	147.5	116.25–191.25	−36.5	−20.04
C-reactive protein (mg/dL)	0.9	0.5–1.85	1.8	0.8–5.2	0.4	50	1.25	0.6–2.7	0.3	36.67

Abs. = absolute; IQR = interquartile range; Rel. = relative.

**Table 5:** Adverse event acute and late toxicities.

	Symptoms	No Events	Grade 1	Grade 2	Grade 3	Grade 4	Grade 5
Acute	Nausea	51 (70%)	8 (11%)	11 (15%)	3 (4%)	0	0
	Vomiting	65 (89%)	8 (11%)	0	0	0	0
	Pain	52 (71%)	11 (15%)	8 (11%)	2 (3%)	0	0
	Fever	67 (92%)	5 (7%)	1 (1%)	0	0	0
Late	Gastritis	66 (90%)	0	3 (4%)	4 (5%)	0	0
	Pancreatitis	71 (97%)	2 (3%)	0	0	0	0
	Cholecystits	73 (100%)	0	0	0	0	0
Up to 3 months after radioembolization	REILD	73 (100%)	0	0	0	0	0
	Skin necrosis	73 (100%)	0	0	0	0	0

REILD = radioembolization-induced liver disease.

## Data Availability

The data presented in this study are available on request from the corresponding author. The data are not publicly available due to data protection regulations.
